# Comparative Study on Different Skin Pruritus Mouse Models

**DOI:** 10.3389/fmed.2021.630237

**Published:** 2021-02-23

**Authors:** Guan Donglang, Liu Tongtong, Chen Dan, Zhu Chan, Wang Changming, Yu Guang, Yang Yan, Tang Zongxiang

**Affiliations:** School of Medicine & Holistic Integrative Medicine, Nanjing University of Chinese Medicine, Nanjing, China

**Keywords:** urushiol, pruritus, animal model, psoriasis, atopic dermatitis, allergic contact dermatitis

## Abstract

The animal model is an important tool to study the mechanism of disease formation. Different animal models of pruritus have been adopted based on the purpose of researchers in the study of the itching mechanism. Although the symptoms of various models are quite different, scratching behavior is a key indicator. Therefore, it is necessary to find an animal model that can quickly induce animal scratching and maintain the stability of scratching behavior. In this study, we compared animal models of pruritus induced by four substances and found that the scratching behavior of mice induced by urushiol not only reached the plateau stage quickly but also showed more stability in the plateau phase than that induced by 2,4-dinitrofluorobenzene, oxazolone, and imiquimod. Meanwhile, in the animal model induced by urushiol, the changes of epidermal thickening and inflammatory cell aggregation were also more obvious. In addition, pruritus induced by urushiol is prevalent all over the world, especially in the United States and Europe, involving outdoor groups such as firefighters, forest loggers, and farmers. Therefore, we believe that the urushiol-induced animal model is an ideal choice for the study of the itch formation mechanism and the development of antipruritic drugs.

## Introduction

Chronic pruritus is an obvious symptom of patients with xeroderma, psoriasis, allergic dermatitis, etc. Pruritus severely influences the quality of life of patients (1). In addition, many systemic diseases are also accompanied by pruritus, including diabetes, chronic kidney disease, and psychiatric disorders (2, 3). Itching-related skin diseases often have different symptoms, including xerosis, eczema, dermatitis, urticarial, and cutaneous pruritus (4, 5). To investigate the mechanism of this chronic itch, multiple animal models are used to simulate pruritic symptoms of clinical patients (6). Different animal models of pruritus have been adopted based on the purpose of the researchers in the study of the itching mechanism. Different mouse models of atopic dermatitis (AD), allergic contact dermatitis (ACD), acetone–ether–water (AEW), and psoriasis are widely used to simulate the symptoms of clinical patients. In the process of establishing different animal models, many chemical compounds are used to induce pruritus, including DNFB, oxazolone, imiquimod, and a mixture of acetone and ether (7, 8). Although the symptoms of various models are quite different, scratching behavior is a key indicator.

AD is a chronic eczematous skin disorder characterized by dry and itchy skin. It is a manifestation of immediate hypersensitivity mediated by immunoglobulin E (IgE); delayed-type hypersensitivity is also involved in the skin reaction of patients (9). Oxazolone and DNFB are usually used to induce AD. Oxazolone is a potent contact allergen in humans, which is usually used to induce an AD model (10, 11). It has been reported that the mechanism responsible for producing the scratching in mice after repeated application with oxazolone resembles that of the itching in patients with AD (12). Oxazolone is also used to induce colitis and respiratory allergy (13, 14). DNFB is an decoupling agent, first used to induce typical skin sensitization of the contact type in guinea pigs (15). AD is recognized as a T helper 2 (Th2)–mediated allergic disease, accompanied by increases in cytokines such as tumor necrosis factor α (TNFα), interleukin 4 (IL-4), and IL-17.

Psoriasis is defined as a chronic inflammatory skin disorder characterized by scaly skin plaques, hyperproliferative keratinocytes, and immune cell infiltration into the skin, often accompanied by itching (16). An imiquimod-induced psoriasis model is used to imitate the symptoms of patients with psoriasis in the clinic (17, 18). It has been reported that disturbances in the innate and adaptive cutaneous immune responses are responsible for the development and sustainment of psoriatic inflammation (19, 20).

Contact dermatitis is defined as an inflammation of the skin induced by direct contact of a substance with the surface of the skin. Skin exposure to irritating substances, including solvents, some chemicals, and cosmetics, often results in red, painful rather than itchy, patches on the involved skin areas (21–23). Urushiol-induced ACD is the most common environmental allergic condition in the world. Urushiol as an pruritogen wildly exists in the *Toxicodendron* (formerly *Rhus*) species—poison ivy, poi-oak, and poison sumac (24). It is mainly distributed in Eastern and Central Asia, Vietnam, Korea, Japan, North America, Africa, Australia, New Zealand, and other countries. Urushiol dermatitis patients often develop an extensive, very itchy vesiculo-bullous rash on an infiltrated base or intermittent rashes, with some lesions resembling erythema multiforme (25, 26). Early urushiol-induced ACD model experiments were done on guinea pigs (27).

Due to the complex mechanism, the research on associated with urushiol dermatitis has remained largely unexplored, and no particularly effective drug has been found. Immune cells including CD8^+^ lymphocytes play a central role in urushiol dermatitis, regulated by CD4^+^ T lymphocyte subpopulations (28). Cytokines including thymic stromal lymphopoietin (TSLP), serotonin (5-HT), IL-33, and endothelin (ET-1) also mediate this allergic reaction (29, 30). It was reported that the most common treatment received was oral steroids. The cost of diagnosis in the emergency department is, on average, five times that in the outpatient setting (24). Therefore, the urushiol-induced model is optimal to study the mechanism of ACD, which can be expected to magnify the prevalence of urushiol dermatitis.

Although the cause of the itch was complicated, there were still some common factors that existed. Multiple immune cells participate in pruritus of the irritated itch model mice, including mast cells and CD4^+^ and CD8^+^ T lymphocytes (31, 32). Like keratinocytes, mast cells are a major source of cutaneous proinflammatory mediators thought to underlie the pathology of pruritus, most importantly IL-33, IL-4, and IL-13 (29, 33, 34). So, these pruritus models have some common features to some extent. Multiple pharmaceuticals could reduce cytokines and chemokines including histamine, TSLP, IL-5, IL-6, IL-8, and IL-13 to relieve the pruritus of the AD induced by DNFB (35, 36). The levels of serum IgE, IL-4, and AD-involved cytokines, such as TNFα, interferon-γ (IFN-γ), IL-1β, TSLP, IL-33, and IL-25, also mediate inflammation in an oxazolone-induced mouse model (19, 37). Different cytokines and chemokines, such as interleukin, TNFα, INFγ, and monocyte chemotactic protein 1 (MCP-1), also increased in imiquimod-induced psoriasis (38, 39).

The aim of this research was to evaluate the skin lesions and behavior of different animal models induced by different irritants. Therefore, the number of inflammatory cytokine immune cells was measured, and the phenotype of the skin was observed.

## Materials and Methods

### Animals

Male C57bl/6J mice 6–8 weeks old were used in the behavioral experiments. All animals were habituated in a room with a 12 h light–dark cycle. The temperature was sustained at 26°C, with humidity at 35%. All mice were kept in the SPF animal center at the Nanjing University of Chinese Medicine. All animal experiments were approved by the animal ethics committee of the Nanjing University of Chinese Medicine.

### Pruritic Models

All mice were divided into five groups, exposed to acetone, urushiol, 2,4-dinitrofluorobenzene (DNFB), oxazolone, and imiquimod. Mice were shaved 5 or 2 days prior. Mice were sensitized with 2.0% (wt/vol) urushiol, DNFB, or oxazolone on the abdominal skin, followed by challenges on the nape of neck 5 days later with 0.5% urushiol, DNFB, or oxazolone. The imiquimod-induced pruritic model was established through painting 5% imiquimod on the neck of mice every day. Urushiol, DNFB, and oxazolone were dissolved in acetone, while imiquimod cream was painted directly. Acetone was used in control mice.

### Materials

DNFB and oxazolone were purchased from Sigma. Imiquimod cream was purchased as a prescription drug (produced by Sichuan Med-shine Pharmaceutical, Co., LTD.). Urushiol was extracted from Chinese lacquer trees (HPLC≥95%) (40). Hematoxylin and eosin (H&E) staining reagent and Giemsa staining reagent were obtained from Solarbio Life Sciences.

### Behavioral Experiments

Mice were habituated in a box with dimensions of 15 × 15 × 15 cm for 15 min every day before experiments. Then, urushiol, DNFB, oxazolone, and imiquimod were painted on the necks of the mice, respectively, shaved 2 days prior. Behaviors of the mice were recorded by video for at least 1 h. We recorded scratching bouts of the mice at 9:00 p.m. every day for 10 days. Scratching numbers were defined as the number of times the hind limbs scratched the neck.

### Histological Analysis

Mice were decapitated quickly on the 10th day. The neck skin of the control and model mice was collected, with an area of 8 × 8 mm. Skin was posterior fixed for 24 h in 4% paraformaldehyde and then placed in 30% sugar for 3–5 days. Skin tissue slices were prepared using a frozen section with a thickness of 15 μm, for histochemical experimentation. H&E staining and toluidine blue staining were used to observe the skin lesions and mast cell. Giemsa staining was used to determine the number of inflammatory cells.

### Statistical Analysis

The results are expressed as mean ± SEM. The significance of the differences between groups was calculated using Student's *t*-test and ANOVA analysis. In all analyses, a *p* < 0.05 is considered to be statistically significant.

## Results

### Four Established Pruritic Mouse Models

**Figure 1:**
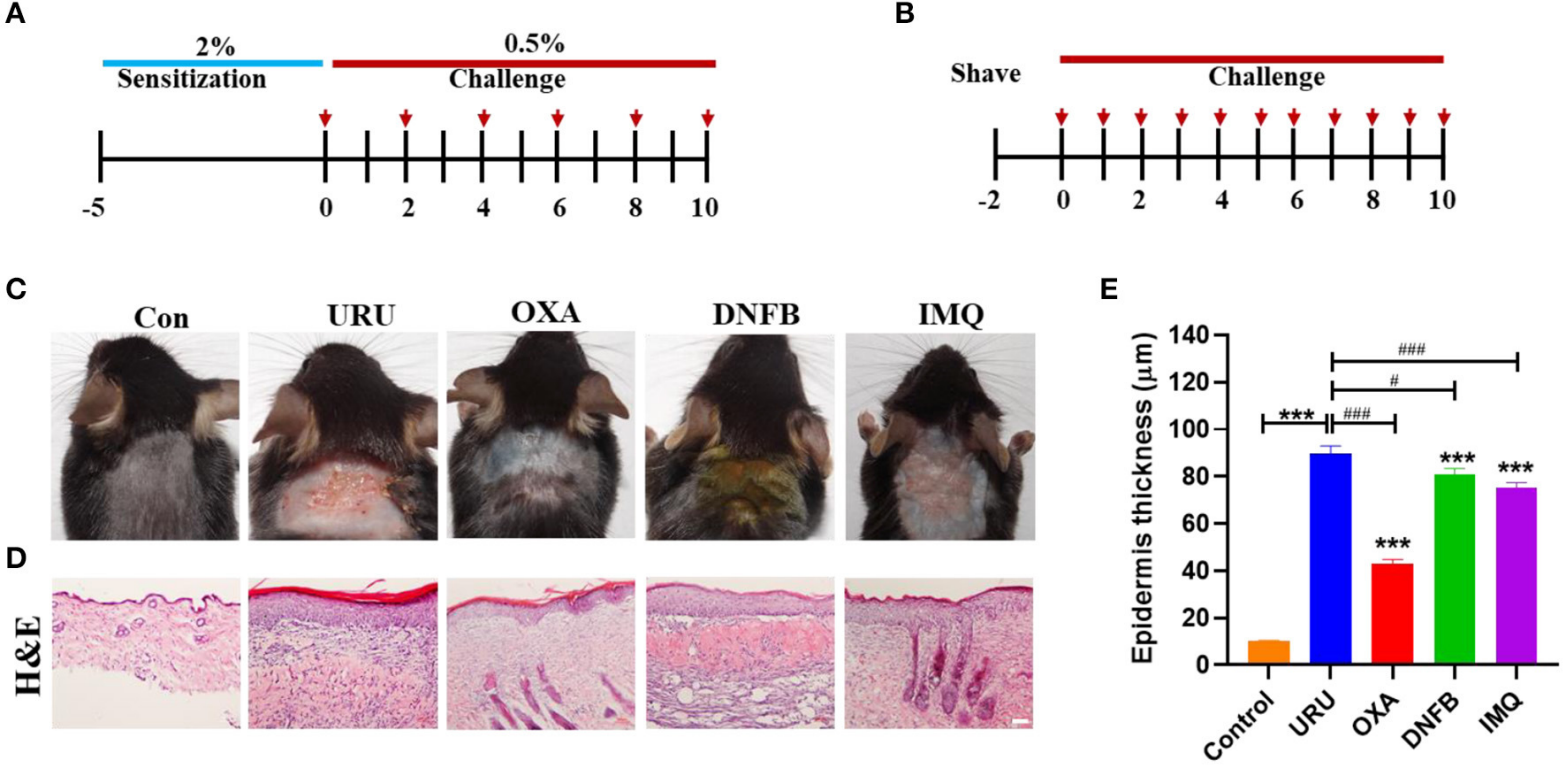
Itchy animals induced by pruritic irritant showed severe skin lesions. **(A)** The itch model was induced by different irritants [urushiol, 2,4-dinitrofluorobenzene (DNFB), and oxazolone]. The red arrow shows the treatment day with the pruritic irritant. **(B)** The psoriasis model was induced by imiquimod. **(C)** Different itch model mice showed obvious skin lesions on the neck compared with control mice. **(D)** Hematoxylin and eosin (H&E) painting of the skin slice on the 10th day. Model mice showed thickening to different degrees. Scale bar 20 μm. **(E)** Statistics of the epidermis thickness of the model mice and control mice. Significant difference is indicated by *p*-value (*compared with control mice; ^#^compared with urushiol-induced model mice). ***p < 0.001, **p < 0.01, *p < 0.05; ^###^*p* < 0.01.

To compare the different pruritic compounds that induced itch, we established four chronic pruritic models, using urushiol, DNFB, oxazolone, and imiquimod on the neck of mice. Mice were sensitized with 2.0% (wt/vol) urushiol, DNFB, or oxazolone on the abdominal skin, followed by challenges on the nape of the neck 5 days later with 0.5% urushiol, DNFB, or oxazolone (Figure 1A). The imiquimod-induced pruritic model was established by painting 5% imiquimod on the neck of mice every day (Figure 1B). Control mice were treated with acetone. Our results showed that all the model mice displayed skin lesions to different degrees compared with control mice (*p* < 0.001) (Figure 1C). Tissue hyperplasia was the typical characteristic of the skin lesions. The thickness of the skin hyperplasia indicated that all the model mice showed skin lesions to different degrees (Figure 1D). Urushiol-induced model mice showed more severe skin damage than the other three mouse models. The epidermis thickness was more obvious compared with other models (uru, 89.8 ± 3.12 μm; oxa, 43.2 ± 1.60 μm; DNFB, 80.8 ± 2.53 μm; IMQ, 75.2 ± 2.24 μm vs. con, 10.1 ± 0.34 μm, *n* = 4 or 5, *p* < 0.001) (Figure 1E). The results indicated that the urushiol-induced model showed the most serious skin lesions.

### The Difference in Scratching Behavior in the Four Mouse Models

**Figure 2:**
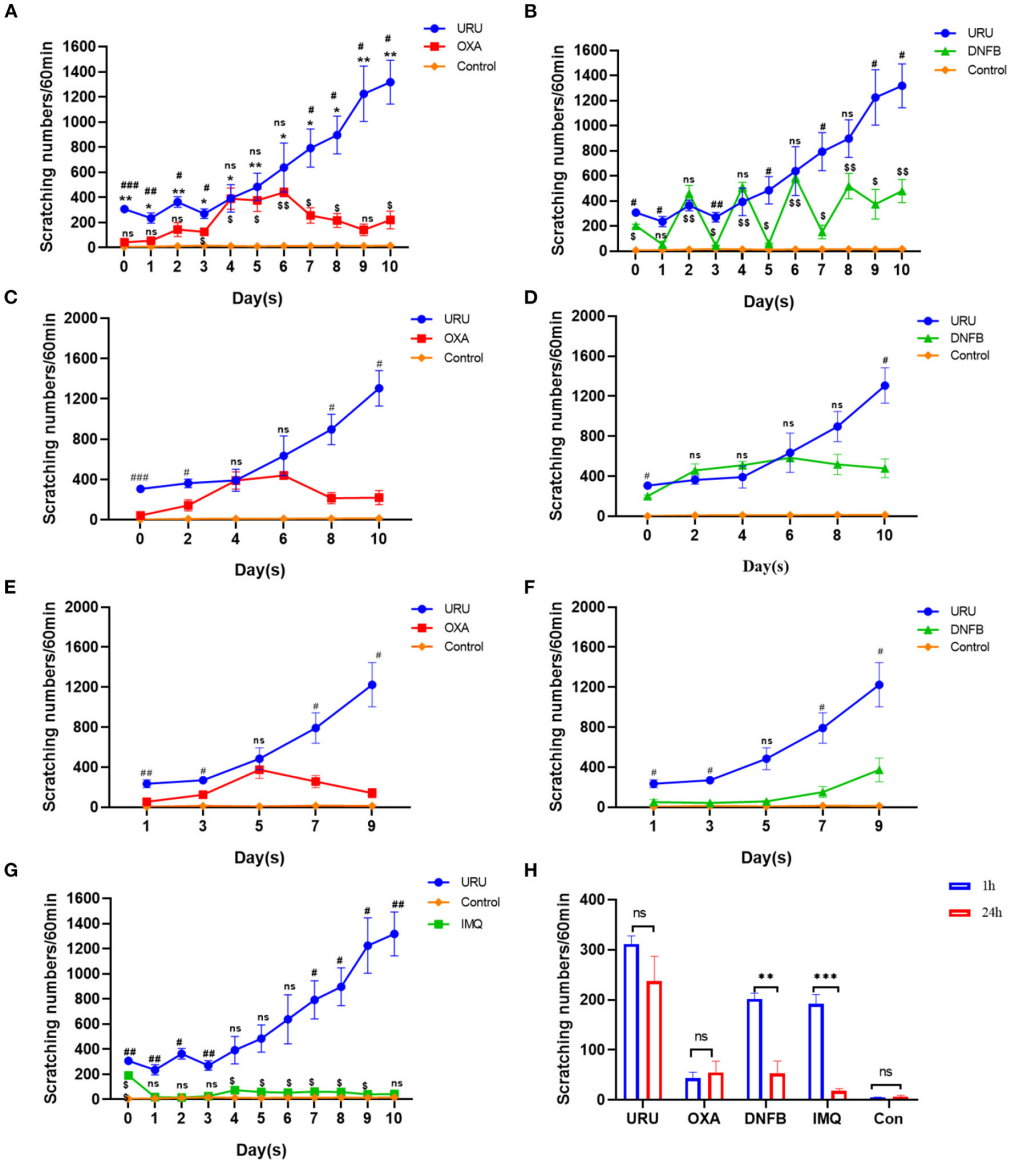
Comparison of scratching bouts between different itch models. **(A)** Scratching bouts of the urushiol-induced mice were higher than those of oxazolone-induced mice and control mice. Significant difference is indicated by *p*-value (*shows urushiol-induced model mice compared with control mice; ^#^shows urushiol-induced model mice compared with oxazolone group; ^$^shows oxazolone group compared with control mice). **(B)** Pruritic behavior of the urushiol-induced mice was more obvious than that of DNFB-induced mice (^#^shows urushiol-induced model mice compared with DNFB group; ^$^shows DNFB group compared with control mice). **(C)** Compared with oxazolone, urushiol induced more obvious pruritus in 1 h (evoked scratching). **(D)** Compared with DNFB, there was no obvious difference in scratching bouts induced by urushiol in 1 h (evoked scratching). **(E)** Compared with oxazolone, urushiol induced more obvious pruritus in 24 h (spontaneous scratching). **(F)** There was an obvious difference in pruritus between DNFB and urushiol model mice. **(G)** Scratching bouts of the urushiol-induced mice were higher than those of IMQ-induced mice (^#^shows urushiol-induced model mice compared with IMQ group; ^$^shows IMQ group compared with control mice). **(H)** Scratching behavior of the different mice shown in 1 and 24 h on the first day. ***p < 0.001, **p <0.01, *p < 0.05; ^##^*p* < 0.01, ^###^*p* < 0.001; ^*$*^*p* < 0.05.

Different animal models were used to imitate the pruritus of patients in clinic, including psoriasis (imiquimod model), xeroderma (AEW model), AD (oxazolone model, DNFB model), and ACD (urushiol model). Pruritus was the obvious symptom of these models. To observe consecutive pruritic behavior, we recorded scratching behavior every day, rather than only at 1 h or 24 h. We found that the four mouse models could show obvious scratching behavior consistent with reported results (8, 17, 29). Except the skin lesions, we found that the scratching bouts of the four mouse models also showed a significant difference. Compared with control mice, all model mice could experience pruritus to different degrees (*p* < 0.01). Urushiol, DNFB, and oxazolone were used to induce ACD. Imiquimod often was used to establish psoriasis, which also showed skin pruritus. Our results showed that urushiol-induced model mice exhibited pruritic behavior earlier than the DNFB, oxazolone, and imiquimod groups (*n* = 4) (Figures 2A,B). Compared with the oxazolone group, urushiol-induced model mice showed more obvious pruritic behavior on the zeroth to third days. The pruritic behavior of the oxazolone-induced model mice reached a plateau on the fourth day (Figures 2A,C,E). DNFB-induced model mice showed serious pruritus in 1 h after application of DNFB. The itchy behavior of the mice in 24 h was reduced until the eighth day (Figures 2B,D,F). Our results showed that imiquimod-induced psoriasis exhibited less severe pruritic behavior in mice than the urushiol group (Figure 2G). Especially, we found that there was no difference in scratching bouts between 1 and 24 h in the urushiol and oxazolone groups on the first day (urushiol, 307 ± 14.3 vs. 235 ± 40.5, *p* = 0.20; oxazolone, 44 ± 11.6 vs. 55 ± 22.4, *p* = 0.67) (Figure 2H). However, there was a significant difference between 1 and 24 h in the DNFB and imiquimod groups (DNFB, 202 ± 11.3 vs. 53 ± 24.7, *p* < 0.01; imiquimod, 192 ± 18.8 vs. 18 ± 4.4, *p* < 0.001). The different irritants inducing pruritus have different pathogenic mechanisms. We speculated that after treatment with different irritants for 1 h, there was direct stimulus to the skin of the model mice. After 24 h, pruritus tends to be stable. DNFB, as a strong sensitizer, also had multiple side effects, such as apoptotic death of skin dendritic cells occurring after exposure and promotion of tumors (41, 42). We concluded that compared with other irritants, urushiol was a relatively stable, safe, and effective irritant for itching.

### Inflammatory Cells of the Irritant Itch Models

**Figure 3:**
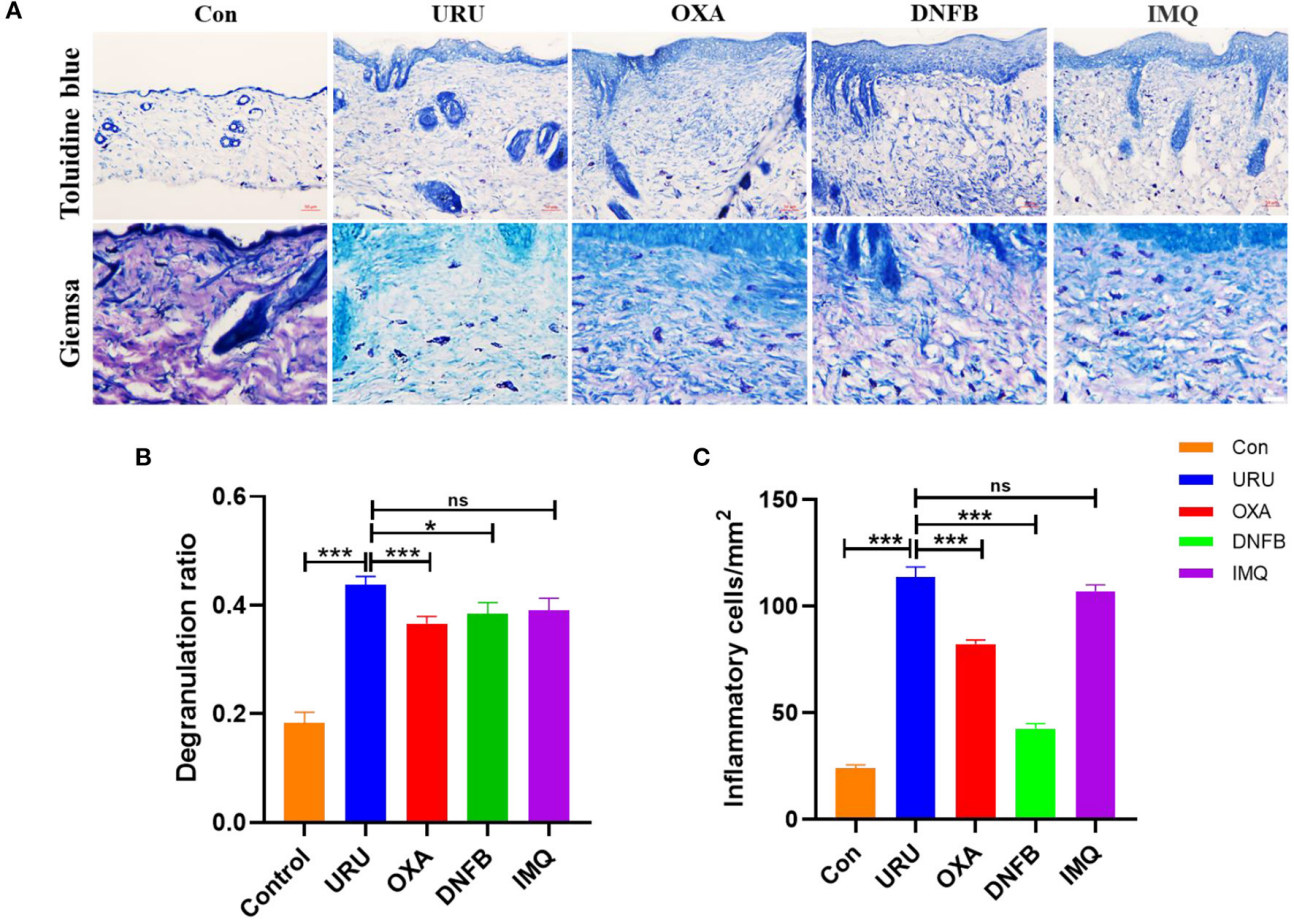
Inflammatory cells mediated itch of the model mice. **(A)** Immunohistochemistry staining of the skin tissue. Toluidine blue staining of the model mice and control mice (upper figure), scale bar, 50 μm. Giemsa staining of the basophils and eosinophils, scale bar 25 μm (bottom of **A**). **(B)** Degranulation ratio of the model mice increased compared to control mice. **(C)** Inflammatory cells in skin tissue of the mice. ***p < 0.001, **p < 0.01, *p < 0.05.

Despite the diversity of the mechanisms in different itch models of mice, inflammatory cells and cytokines have been reported to be involved in the formation of pruritus in mice (43–45). A mast cell is a kind of inflammatory cell, which we have paid close attention to recently. To speculate on whether mast cells participate in the pruritus behavior of these mouse models, we calculated the statistic degranulation percentage of mast cells, using toluidine blue painting. Our results showed that the degranulation percentage of the four mouse models was higher than that of control mice (*p* < 0.001) (Figures 3A,B). Therefore, we speculate that the cytokines and chemokines released by mast cells after degranulation may be the cause of pruritus in the model mice. Other than mast cells, basophils and eosinophils also participate in the itch of these mouse models. Our results showed that both basophils and eosinophils increase obviously compared with control mice (*p* < 0.01) (Figure 3C). To observe the inflammatory cell aggregation induced by different irritants, we compared urushiol-, oxazolone-, DNFB-, and imiquimod-induced itch models. Statistical data revealed that basophil and eosinophil aggregation induced by urushiol was more obvious compared to oxazolone and DNFB (113.6 ± 4.7; oxazolone, 82.14 ± 2.0, *p* < 0.001; DNFB, 42.5 ± 2.2, *p* < 0.001). There was no difference between the urushiol-induced model and the imiquimod-induced model (106.9 ± 2.9, *p* = 0.2037). We believe that different inflammatory cells, including mast cells, basophils and eosinophils, and cytokines, mediated the pruritus of the irritant itch.

## Discussion

Animal models of human disease refer to animals with human disease simulation performance established in various medical science research studies. The use of animal models is a very important experimental method and means in modern biomedical research, which is conducive to more convenient and effective understanding of the occurrence and development of human diseases and research on prevention and control measures. We observed four animal models simulating human pruritus and compared their pruritus characteristics and pathogenesis. It is hoped that these results can provide some references for researchers who are studying different diseases with pruritus.

Pruritus is a disease and a symptom of different diseases. For itch researchers, it is necessary and important to establish an animal model with obvious, stable, and persistent itching behavior. Scratching bouts were usually considered to be an indicator for evaluating itching in mice. Our consecutive record of the behavior showed that the scratching bouts of the urushiol-induced model were more obvious, sustained, and stable than those of DNFB-induced mice. In addition, the pruritus symptom of the urushiol-induced model mice was more severe than oxazolone- and imiquimod-induced model mice. At the same time, urushiol is more likely to induce pruritus than other irritants. Compared with other models, the urushiol-induced model is easier to establish. The characteristics of these animal models can indeed provide a choice for researchers with different directions and who are interested in itching.

The purpose of basic research is to seek effective treatment methods and approaches. The mechanisms of pruritus caused by different diseases are different, but there may be some of the same itching molecules, itch-related receptors, and immune cells in human and animal bodies, which play an important role. For example, IL-22, IL-23, IL-31, and IL-33 were increased in the skin of patients and model animals with allergic dermatitis, AD, and psoriasis (30, 46). In the skin of AD and psoriasis patients, the expression of these receptors such as phospholipase A2, substance P, Nav1.7, and TRPV1 was positively correlated with the degree of skin lesions. In addition, cytokines such as IL-17A, IL-23A, and IL-31 had elevated gene transcript levels in both itchy atopic and psoriatic skin (47). So far, although we are still unable to determine whether the pruritus in different animal models has the same material basis, we can compare and analyze the data obtained in different animal models of pruritus and find out whether there is a certain rule. In any case, the determination of itch targets will effectively promote the development of antipruritic drugs.

## Data Availability Statement

The original contributions presented in the study are included in the article/supplementary material, further inquiries can be directed to the corresponding author/s.

## Ethics Statement

The animal study was reviewed and approved by Animal Ethics Committee of Nanjing University of Chinese Medicine.

## Author Contributions

GD and LT are mainly responsible for data collection and data analysis. CD, ZC, WC, and YG are responsible for review. YY are responsible for data analysis and writing. TZ are responsible for the writing and review. All authors contributed to the article and approved the submitted version.

## Conflict of Interest

The authors declare that the research was conducted in the absence of any commercial or financial relationships that could be construed as a potential conflict of interest.
